# Glioma-Associated Oncogene Homolog Inhibitors Have the Potential of Suppressing Cancer Stem Cells of Breast Cancer

**DOI:** 10.3390/ijms19051375

**Published:** 2018-05-05

**Authors:** Kuo-Shyang Jeng, Chi-Juei Jeng, I-Shyan Sheen, Szu-Hua Wu, Ssu-Jung Lu, Chih-Hsuan Wang, Chiung-Fang Chang

**Affiliations:** 1 General Surgery Division and Department of Medical Research, Far Eastern Memorial Hospital, New Taipei City 220, Taiwan; kevin.ksjeng@gmail.com (K.-S.J.); sunnylu312@gmail.com (S.-J.L.); joyce.walawala@gmail.com (C.-H.W.); 2 Postgraduate Institute of Medicine, National Taiwan University, Taipei City 106, Taiwan; b91401102@ntu.edu.tw; 3 Department of Internal Medicine, Chang-Gung Memorial Hospital, Linkou Medical Center, Taoyuan City 333, Taiwan; happy95kevin@gmail.com; 4 Food Safety, Hygiene and Management University of Birmingham, Birmingham B152TT, UK; SWX781@student.bham.ac.uk

**Keywords:** breast carcinoma, sonic hedgehog signaling pathway, Gli-1, GANT-58, HPI-1

## Abstract

Overexpression of Sonic Hedgehog signaling (Shh) pathway molecules is associated with invasiveness and recurrence in breast carcinoma. Therefore, inhibition of the Shh pathway downstream molecule Glioma-associated Oncogene Homolog (Gli) was investigated for its ability to reduce progression and invasiveness of patient-derived breast cancer cells and cell lines. Human primary breast cancer T2 cells with high expression of Shh signaling pathway molecules were compared with breast cancer line MDA-MB-231 cells. The therapeutic effects of Gli inhibitors were examined in terms of the cell proliferation, apoptosis, cancer stem cells, cell migration and gene expression. Blockade of the Shh signaling pathway could reduce cell proliferation and migration only in MDA-MB-231 cells. Hh pathway inhibitor-1 (HPI-1) increased the percentages of late apoptotic cells in MDA-MB-231 cells and early apoptotic cells in T2 cells. It reduced Bcl2 expression for cell proliferation and increased Bim expression for apoptosis. In addition, Gli inhibitor HPI-1 decreased significantly the percentages of cancer stem cells in T2 cells. HPI-1 worked more effectively than GANT-58 against breast carcinoma cells. In conclusion, HPI-1 could inhibit cell proliferation, reduce cell invasion and decrease cancer stem cell population in breast cancer cells. To target Gli-1 could be a potential strategy to suppress breast cancer stem cells.

## 1. Introduction

Breast carcinoma is the most common cancer among women worldwide [1,2]. The current main treatments include surgery, radiation, chemotherapy, hormone therapy and target therapy [3,4]. However, metastases at distant sites and recurrence are the main causes of death in breast cancer. Breast cancer stem cells (CSC) CD44^+^CD24^low^ exhibit enhanced invasive properties for the metastasis and escape from the therapies [5,6]. Targeting CSC could be a strategy to prevent metastasis and recurrence.

The Sonic Hedgehog signaling (Shh) pathway plays an essential role in maintaining cancer stem cells and side populations [7,8]. This pathway is also involved in breast carcinogenesis, invasion and metastasis [9,10,11,12]. Our previous study suggested overexpression of Hh signaling pathway molecules Shh, Ptch-1, Smo and glioma-associated oncogene homolog (Gli) is associated with invasiveness and could be potential biomarkers for breast cancer recurrence [13]. Serum Shh and interleukin-6 (IL-6) could be useful prognostic biomarkers in progressive breast cancer [14]. Shh promotes glycolysis and proliferation of breast cancer [15]. The hypomethylation in Shh promoter leads to its overexpression in breast cancer patients [16]. Shh overexpression was associated with nuclear factor kappa-light-chain-enhancer of activated B cells (NF-κB) activation, as a poor prognosis indicator for breast cancer [17]. In addition, the Shh pathway mediates progression from a non-invasive phenotype to an invasive phenotype based on the ratio Gli-1 nuclear translocation [18]. Elevated Gli-1 and Gli-2 protein level in human breast cancer was associated with poor prognosis and progressive stages of disease [19,20]. Gli-1 is crucial for hypoxia-induced epithelial-mesenchymal transition (EMT) and invasion of breast cancer [21]. Activated Hh could increase Gli expression and enhance SRY-box 2 (Sox2) and Octamer-binding transcription factor 4 (OCT4) expression to regulate cancer stem cell maintenance [22]. Moreover, chemotherapy could activate Hh-Gli signaling and chemotherapy such as doxorubicin, cisplatin resistance as was mediated by Gli [23,24]. The crosstalking and activation of Shh pathway and Wnt signaling pathway is associated with poor outcomes in breast cancer (BC) patients as well as cancer stem cells [25,26]. Cancer stem cells contribute to chemotherapy resistance [27,28]. Therefore, Shh signaling pathway molecules could be potential therapeutic targets in breast cancer [29,30].

Two Gli antagonists GANT58 and HPI-1 were used to inhibit Shh signaling pathway in this study. GANT58 interfered with GLI1 and GLI2-mediated transcription in a dose-dependent manner and reduced growth of prostate cancer [31]. GANT58 induced apoptosis in T leukemia cells by altering Hedgehog signaling pathway [32]. In addition, small molecule Hh inhibitors (HPI-1, 2, 3 and 4) were identified to perturb Gli processing, stability, localization and function [33]. Nanoparticles with HPI-1 inhibit the growth or metastasis in orthotopic model of human pancreatic cancer and hepatocellular carcinoma [34,35].

The therapeutic effects of Gli inhibition remain unclear in primary breast cancer cells. In this study, primary human BC cells with highly expression of Hh signaling molecules were isolated in comparison with BC cell line MDA-MB-231 cells. Besides, two kinds of small-molecule Gli antagonists GANT-58 and HPI-1 were used to compare their therapeutic effects in terms of cell viability, apoptosis, cell migration and cancer stem cells.

## 2. Results

### 2.1. Cell Morphology and Her2 Expression of Breast Carcinoma Cells

**Figure 1:**
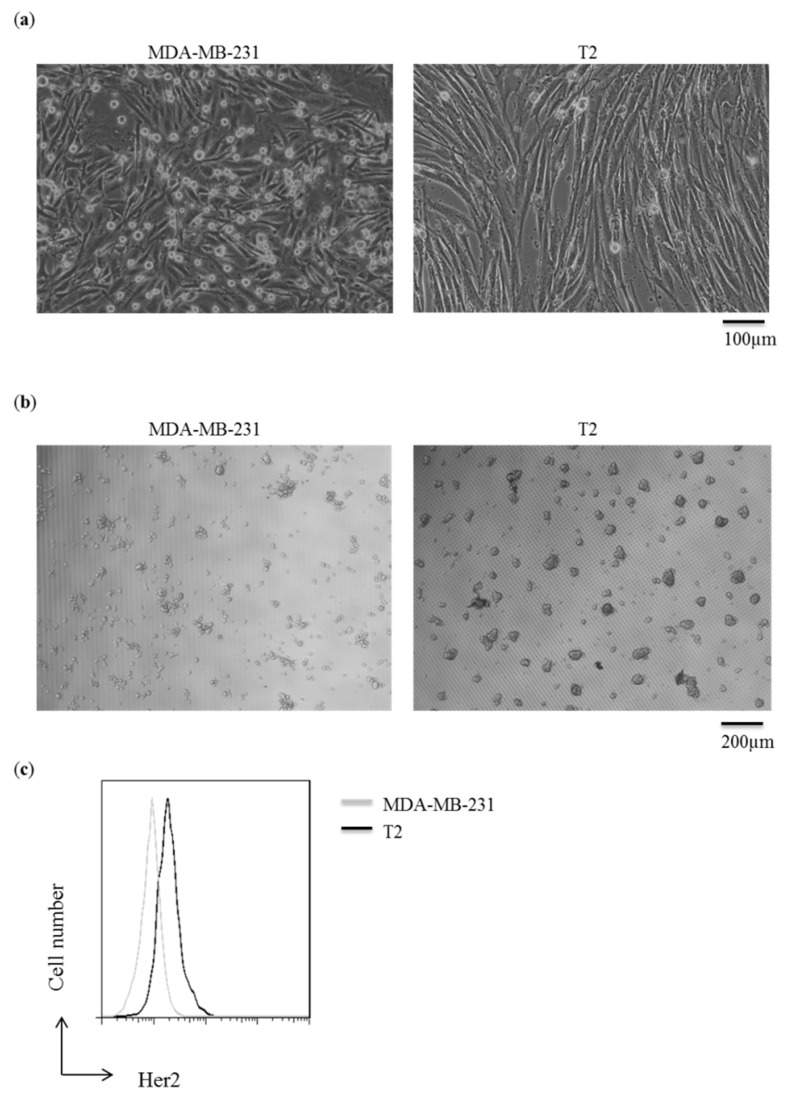
Morphology of breast carcinoma MDA-MB-231 and primary human breast carcinoma T2 cells. (**a**) Cells were cultured in L15 or Dulbecco’s Modified Eagle Medium (DMEM) supplemented with fetal bovine serum (FBS) in a 24 well plate; (**b**) Cells were cultured with the same medium in NanoCulture 24-well plate. All images were taken by an optical microscope; (**c**) Cells were stained with Fluorescein isothiocyanate (FITC)-labeled Her2 antibody. Data were collected by a FACS Calibur and analyzed by FlowJo software. The *X*-axis represented Her2 expression and *Y*-axis represented the cell number. The results were repeated from three-independent experiments and the representative plot was shown.

The morphology of MDA-MB-231 cells and T2 are similar as epithelial cell subtype and adherent cells (Figure 1a). However, T2 cells can form a bigger spheroid than MDA-MB-231 cells does in 3-dimensional culture (Figure 1b). Breast carcinoma T2 cells were isolated from a patient with estrogen receptor (ER) (−), progesterone receptor (PR) (−) and Her2 (+). Therefore, T2 cells were compared with established breast carcinoma cell line MDA-MB-231 cells with ER (−), PR (−) and human epidermal growth factor receptor (HER) (0−1+). The expression of Her2 was higher in T2 cells than in MDA-MB-231 cells (Figure 1c).

### 2.2. High Expression of Shh Pathway Molecules in Primary Breast Carcinoma

**Figure 2:**
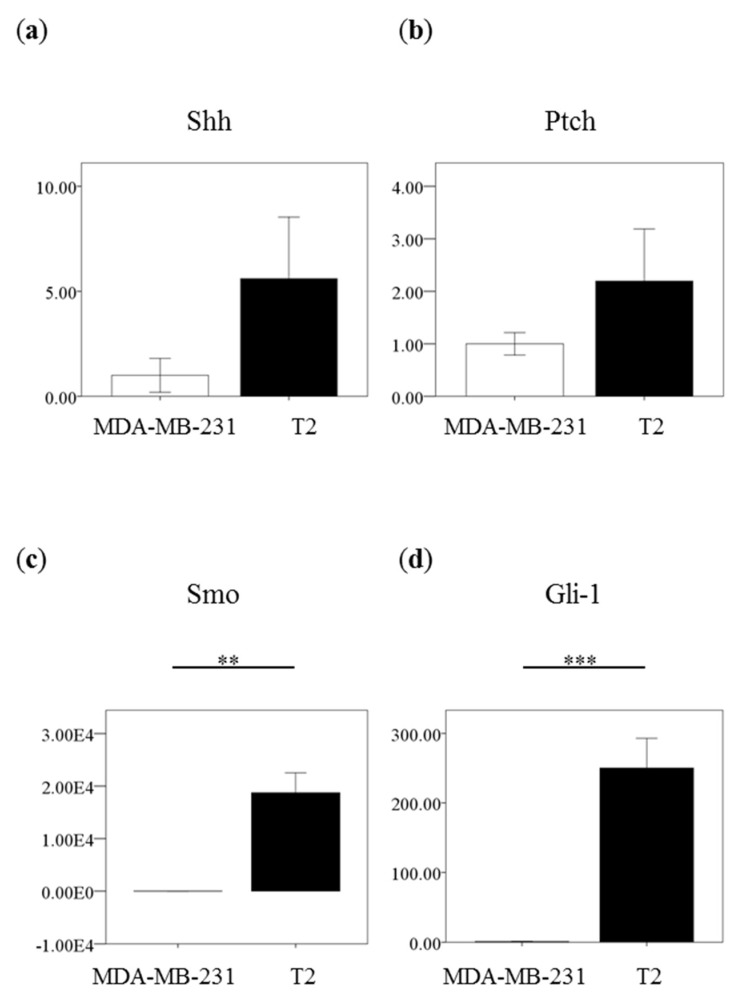
Expression of Shh pathway molecules is higher in primary human breast carcinoma T2 cells than in breast cancer cell line MDA-MB-231. MDA-MB-231 and T2 cells were subjected to quantitative PCR for the gene expression of Shh pathway molecules (**a**) Shh, (**b**) Ptch, (**c**)Smo and (**d**) Gli-1 from 3-independent experiments. (*n* = 3). ** *p*<0.01, *** *p*<0.001.

Shh signaling pathways play an important role in the progression of breast carcinoma. Shh pathway molecules Shh, Ptch-1, Smo and Gli-1 were expressed in MDA-MB-231 and T2 cells. T2 cells express higher Shh pathway molecules than MDA-MB-231 cell line (Figure 2). There were no significant differences between in Shh and Ptch expression in these two cells (Shh, *p* = 0.059; Ptch-1, *p* = 0.112) (Figure 2a,b). However, T2 cells significantly expressed higher mRNA level of Smo and Gli-1 than MDA-MB-231 cells (Smo, *p* = 0.001; Gli, *p* = 0.0005) (Figure 2c,d). It suggested T2 cells could have a stronger Hh signaling pathway via transcription factor GLI-1 than MDA-MB-231 cell line.

### 2.3. Reduced Cell Viability by Gli Inhibitors

**Figure 3:**
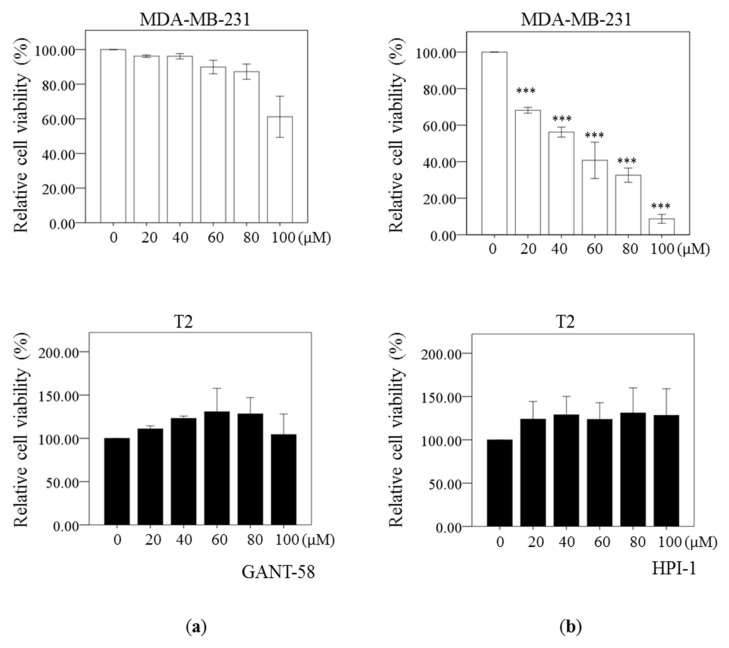
Gli inhibitor HPI-1 (Hh pathway inhibitor, HPI) could reduce the cell viability in breast cancer cell line MDA-MB-231 cells. The cell viability of human breast cancer cell line (MDA-MB-231) and primary human breast carcinoma T2 cell were assessed by 3-(4,5-dimethylthiazol-2-yl)-2,5-diphenyltetrazolium bromide (MTT) assay after 72 h of treatment with Dimethyl sulfoxide (DMSO) (0 μM), GANT-58 (Panel (**a**)) or HPI-1 (Panel (**b**)). The percentages of viable cells treated with a compound or the vehicle control was calculated by normalizing the optical density (O.D.) value to that of the untreated control cultures. The results are represented as the mean ± standard deviation (SD) of three independent experiments performed in triplicate. (*** *p* ≤ 0.001, compared with the cultured treated with DMSO).

The cell viability of human breast cancer cells MDA-MB-231 and T2 was evaluated by 3-(4,5-dimethylthiazol-2-yl)-2,5-diphenyltetrazolium bromide (MTT) assay after exposing the cells to Dimethyl sulfoxide (DMSO) (control), GANT58 or HPI-1 for 72 h. GLI inhibitor GANT-58 could start to reduce the cell proliferation of MDA-MB-231 cells at the highest dose of 100 μM (Figure 3a, upper panel). However, there were no significant differences in the cell viability of T2 cells in absence or presence of GANT-58 (Figure 3a, bottom panel), even at the dose of 100 μM. In addition, another Gli inhibitor HPI-1 decreased cell viability in dose dependent way in MDA-MB-231 cells (Figure 3b, upper panel). However, there were no significant differences in the cell viability of T2 cells in absence or presence of HPI-1 (Figure 3b, bottom panel). Overall, our results demonstrated that HPI-1 is more effective than GANT-58 in reducing the cell viability of breast cancer cells. Both Gli inhibitors at dose of 100 μM cannot reduce the cell viability in T2 cells.

### 2.4. Gli Inhibitors Increased the Percentages of Late Apoptotic Breast Carcinoma Cells

**Figure 4:**
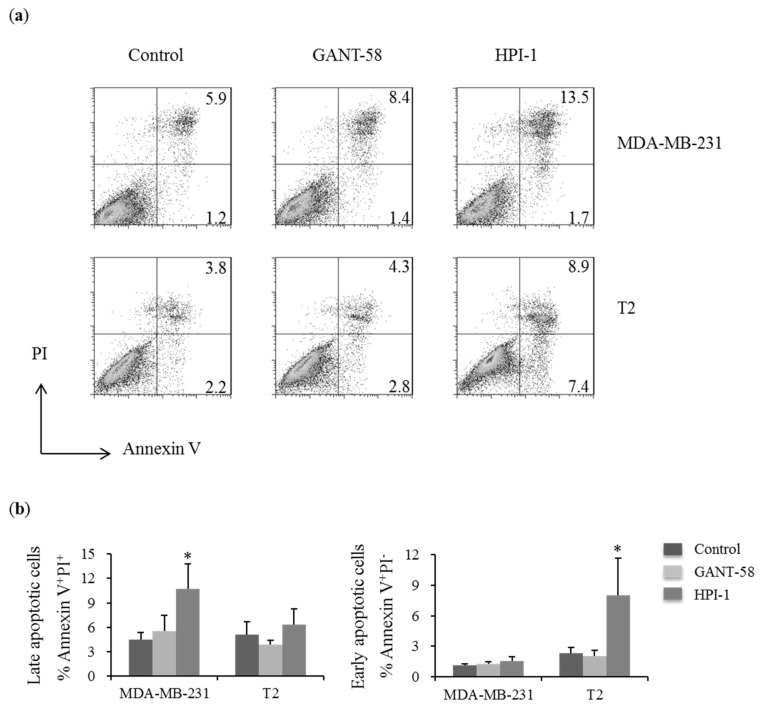
Inhibition of Hh pathway increased the percentages of apoptotic cells in breast cancer cells. (**a**) The apoptosis of human breast cancer cell line (MDA-MB-231) and primary human breast carcinoma T2 cell were assessed by Annexin V and propidium iodide (PI) staining after 48 h of treatment with DMSO (Control), 40 μM GANT-58 or 40 μM HPI-1. The lower right quadrant (Annexin V+/PI−) was considered as early-stage apoptotic cells, the upper right quadrant (Annexin V+/PI+) was considered late-stage apoptotic cells. The percentages of early or late-stage apoptotic cells were shown; (**b**) The mean percentage of apoptotic cells were represented as the mean ± SD of five independent experiments. (* *p* ≤ 0.05, compared with the cultured treated with DMSO, Control).

Increasing the apoptotic cells could lead to the reduction of cell proliferation. Therefore, the effects of Gli-1 inhibitors for the apoptosis were assessed. In MDA-MB-231 cells, HPI-1 increased the percentages of late apoptotic cells (Annexin V+PI+) (Figure 4a upper panel and 4b). In T2 cells, HPI-1 increased the percentages of early apoptotic cells (Annexin V+PI−) (Figure 4a bottom panel and Figure 4b). GANT58 did not alter the percentages of the apoptotic cells (Figure 4a,b). It suggested HPI-1 worked more effective than GANT-58 to induce the apoptosis of breast carcinoma cells.

### 2.5. Decreased the Percentages of Cancer Stem Cells CD44^+^CD24^low^ in Primary Breast Carcinoma Cells

**Figure 5:**
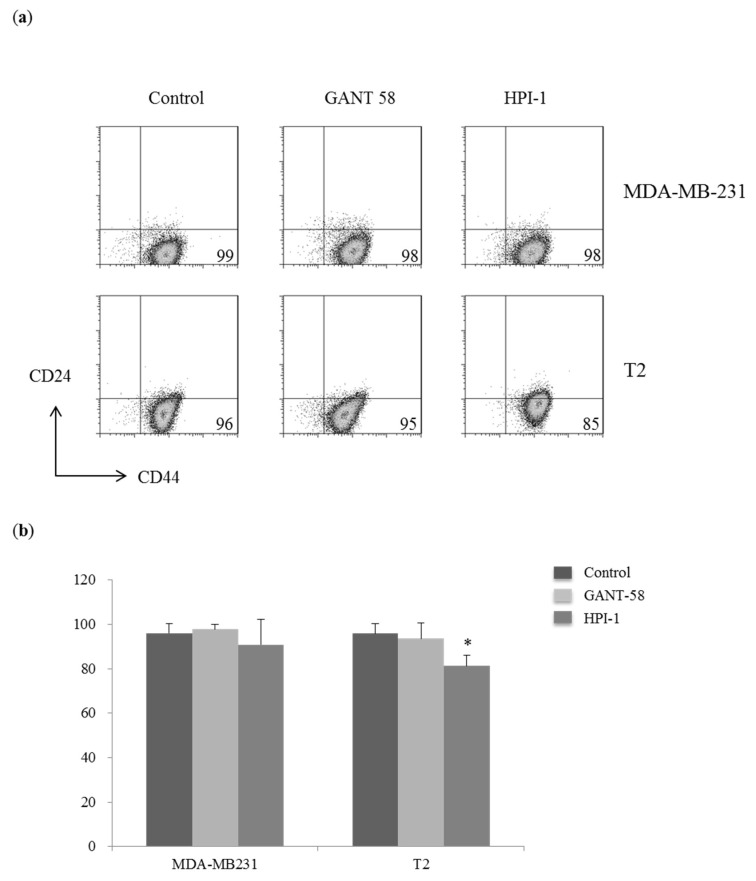
Gli inhibitor reduced the percentages of cancer stem cells (CD44^+^CD24^low^) in T2 cells. (**a**) Human breast cancer cell line (MDA-MB-231) and primary human breast carcinoma T2 cells were collected after 48 h of treatment with DMSO (control), 40 μM GANT-58 or 40 μM HPI-1. Cells were stained with fluorescent antibodies against CD44 and CD24 for cancer stem cells. Data were collected by a FACS Calibur and analyzed by FlowJo software. The lower right quadrant (CD44^+^CD24^low^) was considered as cancer stem cells; (**b**) The mean percentage of cancer stem cells were represented as the mean ± SD of five independent experiments. (* *p* < 0.05, compared with the cultured treated with DMSO, control).

Shh pathway is involved in stem cell regeneration and maintenance. CD44^+^CD24^low^ population is considered as stem cells of breast carcinoma. Both MDA-MB-231 cells and T2 cells have a major population of cancer stem cells CD44^+^CD24^low^. There is no significant change in the expression of CD44 and CD24 in MDA-MB-231 cells with Gli inhibition (Figure 5a, upper panel). In T2 cells, HPI-1 could significantly decrease in the percentage of CD44^+^CD24^low^ cells whereas GANT-58 did not alter the percentage of cancer stem cells (Figure 5a, bottom panel and Figure 5b). Therefore, it suggested HPI-1 could alter the expression of stem cell marker CD24 in T2 cells.

### 2.6. Gli Inhibitor Reduced Migration in Breast Carcinoma Cell Line MDA-MB-231 Cells

**Figure 6:**
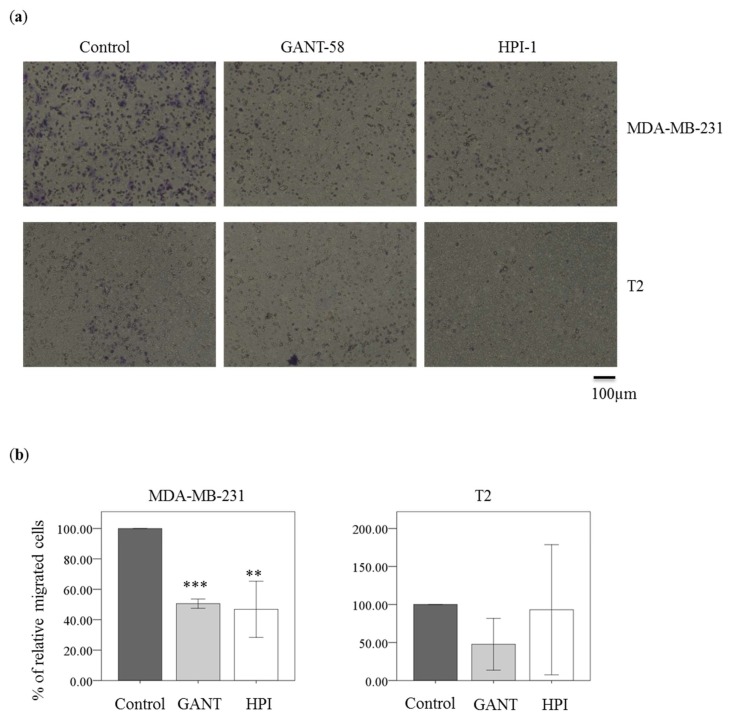
Gli inhibitor reduced cell migration of breast carcinoma cells. (**a**) MDA-MB-231 cells or T2 cells were treated with either DMSO (Control), 40 μM GANT-58 (GANT) and 40 μM HPI-1 (HPI) for 24 h. Magnification: 100×; (**b**) The migrated cells were normalized to vehicle condition as 100% in each experiment. The *y*-axis represented the relative migrated cells counted under 5 different fields from 3 independent experiments. (** *p* < 0.01; *** *p* < 0.001, compared with the cultured treated with DMSO, Control).

Hedgehog signaling pathway molecule Gli-1 is important for migration and invasion of breast cancer. The migrated MDA-MB-231 cells were decreased by GANT-58 or HPI-1 treatment (Figure 6a, upper raw). There was a significant difference between control and HPI-1 or GANT-58 treatment in MDA-MB-231 cells (Figure 6b, left panel). In T2 cells, GANT-58 and HPI-1 could reduce the migration (Figure 6a, bottom raw). However, the differences between control and GANT-58 or HPI-1 treatment were not significant (Figure 6b, right panel). Overall, Gli inhibition could reduce migration in the breast cancer cell line, but not in T2 cells.

### 2.7. Gli inhibition Decreased Bcl2 and MMP2 Expression and Increased Bim Expression

**Figure 7:**
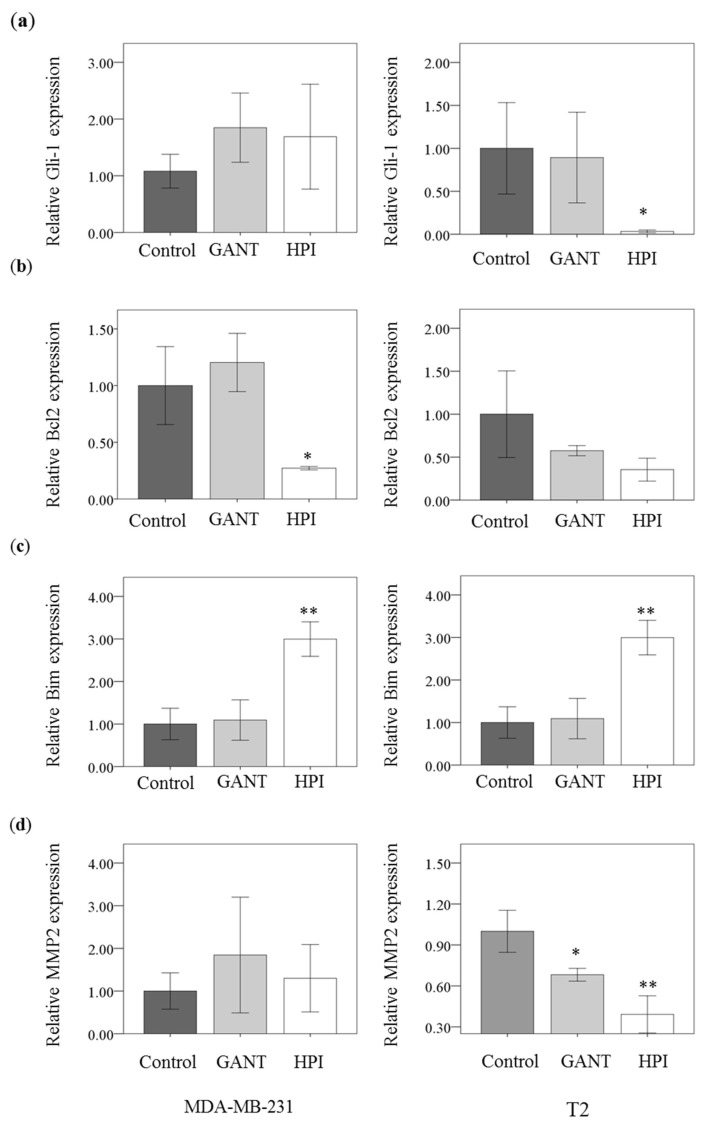
Gli inhibition decrease Bcl2 expression and increase Bim expression. The gene expression of human breast cancer cell line (MDA-MB-231) and primary human breast carcinoma T2 cell were assessed by qPCR after 48 h of treatment with DMSO (Control), 40 μM GANT-58 (GANT) or 40 μM HPI-1 (HPI). The mean gene expression of (**a**) Gli (**b**) Bcl2 (**c**) Bim (**d**) MMP-2 was represented as the mean ± SD of three independent experiments. (* *p* < 0.05; ** *p* < 0.01, compared with the cultured treated with DMSO, control).

Gli inhibitor HPI-1 could reduce the gene expression in Gli-1 (Figure 7a). However, there was no significant difference in Gli-1 expression in presence or absence of GANT-58 treatment. HPI-1 could reduce cell proliferation and induce cell apoptosis. Therefore, the survival gene Bcl2 and apoptotic gene Bim were analyzed with or without the Gli inhibition. HPI-1 could reduce Bcl2 expression only in MDA-MB-231 cells (Figure 7b). HPI-1 could increase Bim expression in both MDA-MB-231 cells and T2 cells (Figure 7c). Matrix Metallopeptidase 2 (MMP2) activity is responsible for cancer cell migration. In T2 cells, GANT-58 and HPI-1 treatment could reduce the expression of MMP2 (Figure 7d). Therefore, Gli inhibition could reduce cell migration of breast cancer cells.

## 3. Discussion

The primary breast cancer T2 cells were successfully isolated and cultured. Breast cancer cell line MDA-MB-231 was used to compare with T2 cells because they shared some similarity such as ER (−), PR (−) and the morphology in a two-dimensional culture system. They also form a major population of cancer stem cells CD44^+^CD24^low^. However, there were some different characters and gene expression in T2 cells and MDA-MB-231 cells. MDA-MB-231 cells grow better without CO_2_, but T2 cells could grow under 5% CO_2_. In 3 dimensional cultures, spheroid formation is as an indicator for drug screening and cancer stem cells [36]. MDA-MB-231 cells could form looser spheroids compared to other breast cancer cell lines [37]. T2 cells could forms multiple spheroids better than MDA-MB-231 cells. It suggested T2 cells tend to aggregate and form clusters.

T2 cells have higher expression of Shh signaling pathway molecules than MDA-MB-231 cells. MDA-MB-231 cells express relative low Gli but express moderate Shh molecules among different cell lines [17,38]. Therefore, it suggested that Hh molecules were overexpressed in T2 cells, especially the downstream molecule Gli-1. Our previous data suggested that overexpression of Shh signaling pathway molecules were correlated with invasiveness and recurrence in breast carcinoma [13]. Gli expression was correlated with Her2 expression whereas Shh expression was correlated with tumor grade [39]. In this study, T2 cells express slightly higher Her2 than MDA-MB-231 cells. However, the migration activity of T2 cells seems lower than MDA-MB-231 cells, which could be due to the phenotype of T2 cells.

The advantages of Gli inhibition is to bypass the Smo mutations and directly inhibit the Hh signaling downstream transcription factor [40]. Moreover, osteopontin (OPN) regulates Gli-1 activity, expression and induces epithelial-mesenchymal plasticity in breast carcinoma [23]. Therefore, inhibition of Gli could be a better strategy. Two Gli antagonists GANT58 and HPI-1 were used in this study. GANT58 demonstrates a high degree of selectivity for Hh/Gli signaling [31]. HPI-1 could reduce tumor growth of CD133-expressing hepatocellular carcinoma [35]. However, it has not been compared the therapeutic efficacy of GANT58 and HPI-1 in breast carcinoma with higher or lower expression of Hh molecules.

Our study demonstrated Gli inhibition could induce apoptosis and reduce survival in both breast carcinoma cells although the Gli expression was different in these two cell lines. Gli inhibitor HPI-1 could significantly reduce the expression of survival protein Bcl2 and increase apoptotic protein Bim in MDA-MB-231 cells. The proapoptotic protein Bim binding to the antiapoptotic Bcl2 results in the release of Bax/Bak to promote apoptosis. Therefore, it is correlated with the increased apoptotic cells in MDA-MB-231 cells. In T2 cells, increased Bim expression could be associated with increased apoptotic cells. Our study supported HPI-1 treatment could alter the gene expression of Bcl2 and Bim to induce apoptosis in breast cancer cells. It is also true that Gli inhibitor GANT-61 treatment could also decreased Bcl2 and increased proapoptic factor Bax in other breast cancer cell lines MCF and MDA-MB-453 [39]. Moreover, it agreed with previous study that inhibition of Hh pathway by itraconazole could induce apoptosis, reduce Bcl2 protein expression and autophagic cell death in breast cancer cells [41]. However, the cell viability of T2 cells was less sensitive to Gli-1 inhibition whereas HPI-1 reduced the cell viability of MDA-MB-231 cells in a dose-dependent manner. It may require higher doses of Gli antagonists because of Gli-1 overexpression in T2 cells. It is possible that the concentration of Gli inhibitor could be too high to be specific. Previous study demonstrated that the IC50 of HPI-1 was about 1~6 μM in HEK293T cells but it was over 30 μM in Wnt-LIGHT cells (Wnt3a) HEK293T cells [33]. In addition, human hepatocellular carcinoma (HCC) cell line Huh7 cells was treated with 40 μM NanoHHI (HPI-1 in nanoparticle) to inhibit cell growth and reduce 40% cell viability [35]. It suggested that the IC50 of HPI-1 for some cells could be over 30 μM. Therefore, the similar concentration ranges of HPI-1(0~100 μM) was used in this study.

Breast CSC cells (CD44^+^CD24^low^) express stem cell associated genes such as Oct4, Aldh1, Notch, and so on. [42]. CSC cells are highly resistant to chemotherapeutic reagents. Both MDA-MB-231 cells and T2 cells express a major population of cancer stem cells CD44^+^CD24^low^. GANT-58 did not alter cancer stem cell population in both cells but HPI-1 could reduce the percentage of cancer stem cells in T2 cells. HPI-1 work more effectively than GANT-58 in the same dosage. Gli inhibition could reduce significantly in the cell migration of MDA-MB-231 cells, but not in T2 cells with lower ability for the cell migration. MMP2 secretion and activation is associated the metastasis of breast carcinoma [43]. Although there were no significant differences in MMP2 in MDA-MB-231 cells, it was significantly down-regulated in T2 cells. It could be due to different expression of MMP-2 and MMP-9 in these two types of breast cancer cells.

Among the other antagonists of the Shh signaling pathway, the smo inhibitor cyclopamine could reduce cell proliferation, induce apoptosis, alter cell cycle and decrease invasive ability in breast cancer cells [44,45,46]. It could reduce cell proliferation of breast cancer stem cells [47]. Other Hh antagonists include cyclopamine, GDC-0449, and so on [29,48,49]. Smo antagonist GDC-0449 is a for basal cell carcinoma (BCC) [50,51]. Recent study suggested that Gli antagonist GANT61 work more effectively than Smo antagonist GDC-0449 (Food and Drug Administration (FDA) approval drug) to reduce cell growth, alter cell cycle in vitro and in vivo [39]. GANT61 could significantly reduce cell proliferation, cell motility and invasion, decrease CSC proportion and mammospheres [12,52,53]. In addition, antibodies blockade of Hh signaling in peri-tumoral stromal cells could be a therapeutic approach in basal-like breast cancers [54,55].

## 4. Materials and Methods

### 4.1. Cell Culture

MDA-MB-231 cells were purchased from Bioresource Collection and Research Center, Taiwan. Cells were cultured in L15 (Hyclone, South Logan, UT, USA) containing 10% fetal bovine serum (FBS), 100 unit/mL penicillin, 100 mg/mL streptomycin and 0.1 mM NEAA (Gibco, Carlsbad, CA, USA) in a humidified atmosphere without CO_2_ at 37 °C. For primary cell culture from the patient’ sample, all the procedures were approved by the Medical Ethics Committee of Far Eastern Memorial Hospital (the approval number: 099117-F, 16 May, 2011). A signed consent from was obtained from the patient who underwent primary surgical resection and enrolled in this study. Breast carcinoma T2 cells were isolated from a patient with ER (−), PR (−) and Her2 (+). Primary cells were cultured in DMEM medium (Gibco, Carlsbad, CA, USA) containing 20% fetal bovine serum (FBS), 100 unit/mL penicillin, 100 mg/mL streptomycin and 0.1 mM Non-essential Amino Acid (NEAA) (Gibco) in a humidified atmosphere of 5% CO_2_ at 37 °C.

### 4.2. RNA Isolation and Quantitative Real-Time PCR

Total RNA was isolated from breast carcinoma cells by with the RNAspin Mini RNA isolation kit (GE Healthcare, Little Chalfont, UK) according to the manufacture’s protocol. The total RNA was reversely transcripted to cDNA by a high capacity cDNA reverse transcription (RT) kit (Applied Biosystems, Foster City, CA, USA). The mRNA expression was analyzed by a real-time polymerase chain reaction (PCR) machine Roche LightCycler480 (Roche Applied Science, Mannheim, Germany). The PCR mixture were prepared with cDNA template, forward and reverse primers and SYBR Green Master Mix. For real-time PCR, procedures were as follows: hot start at 95 °C for 1 min, followed by 45 cycles of denaturing at 95 °C for 10 s, annealing at 58 °C for 5 s and extension at 72 °C for 20 s. PCR products were detected using 2% agarose gel to confirm the expected sizes. The following specific primer pairs for Shh, Ptch-1, Gli-1, Smo, Bcl2, Bim, MMP2 and GAPDH are summarized in the Table 1. Gene expression was analyzed after normalization to control gene GAPDH. The relative gene expression was calculated by 2 − ∆∆Ct method. ∆∆Ct = ∆Ct (target gene) − ∆Ct (control, GAPDH), relative gene expression = 2 − ∆∆Ct.

### 4.3. Cell Viability Assay

The cell viability was determined by MTT assay. Cells were seeded into 96-well plate (1 × 10^4^ cells/well) and treated with GANT-58 (Millipore, Temecula, CA, USA) or HPI-1 (Tocris Bioscience, Abingdon, UK) for 48 h MTT (Sigma Aldrich, St. Louis, MO, USA) was added to each well and incubated for last 4 h. The medium with MTT was aspirated, followed by the addition of DMSO (Sigma). The absorbance 570 nm was measured by an ELISA reader (Bio-Rad, Hercules, CA, USA).

### 4.4. Flow Cytometry

For apoptosis assay, cells were stained with Fluorescein isothiocyante (FITC) Annexin V Apoptosis Detection Kit I (BD Pharmingen, San Jose, CA, USA) according to the manufacturer’s instructions. For cancer stem cells, cells were stained with anti-CD44 antibody labeled with FITC and anti-CD24 antibody labeled with Allophycocyanin (APC) (Biolegend, San Diego, CA, USA). The data were collected on a FACS Calibur (BD Biosciences, San Jose, CA, USA) and analyzed using FlowJo software (Tree Star Inc., Ashland, OR, USA).

### 4.5. Cell Migration Assay

8-μm-pore Transwell inserts were used for the cell migration assay (Corning Inc., Tewksbury, CA, USA). Breast carcinoma cells was seeded at the density of 1 × 10^5^ cells/well in the transwell containing L15 medium with 1% FBS (for MDA-MB-231 cells) or DMEM medium with 2% FBS (for T2 cells) and the transwell was placed in 24-well containing L15 medium with 10% FBS or DMEM medium with 20% FBS. Cells were treated with 40 μM Gli inhibitor GANT58 or HPI-1 for 24 h. The migrated breast carcinoma cells were evaluated 24-h post-incubation at 37 °C. The migrated cells were fixed with 10% formaldehyde and washed with PBS. The migrated cells were then stained with crystal violet (Sigma Aldrich) for 2 h and washed with sterile ddH_2_O. Images (100×) were collected under a Leica microscope (Wetzlar, Germany).

### 4.6. Statistical Analysis

Comparisons among groups were made using SPSS (Chicago, IL, USA). All the data were reported as mean ± SD. Comparisons between different groups for each point were performed using the one-way analysis of variance (ANOVA). All tests were 2-tailed, and *p* < 0.05 were considered statistically significant.

## 5. Conclusions

Gli inhibitor HPI-1 could reduce cell proliferation, induce apoptosis, alter cancer stem cell expression and reduce cell migration in breast cancer cells. Targeting Gli-1 could be a potential strategy to suppress breast cancer stem cells.

## Figures and Tables

**Table 1:** Primers sequences for quantitative PCR.

Genes	Primer sequence	Amplicon (bp)
Shh	Forward: 5’- GAAAGCAGAGAACTCGGTGG-3	170
	Reverse: 5’-GGAAAGTGAGGAAGTCGCTG-3’	
Ptch-1	Forward: 5’-CTCCCAAGCAAATGTACGAGCA-3’	148
	Reverse: 5’-TGAGTGGAGTTCTGTGCGACAC-3’	
Smo	Forward: 5’- GGGAGGCTACTTCCTCATCC-3	167
	Reverse: 5’- GGCAGCTGAAGGTAATGAGC-3’	
Gli-1	Forward: 5’-CTCCCGAAGGACAGGTATGTAAC-3’	248
	Reverse:5’-CCCTACTCTTTAGGCACTAGAGTTG-3’	
Bcl2	Forward: 5’-CTG GTG GAC AAC ATC GC-3’	135
	Reverse: 5’-GGA GAA ATC AAA CAG AGG C-3’	
Bim	Forward: 5’-TAAGTTCTGAGTGTGACCGAGA-3’	96
	Reverse: 5’-GCTCTGTCTGTAGGGAGGTAGG-3’	
MMP2	Forward: 5’-GATACCCCTTTGACGGTAAGGA-3’	112
	Reverse: 5’-CCTTCTCCCAAGGTCCATAGC-3’	
GAPDH	Forward: 5’-CACCACCAACTGCTTAG-3’	334
	Reverse: 5’-CTTCACCACCTTCTTGATG-3’	

## Abbreviations

Shh	Sonic hedgehog
Gli	Glioma-associated oncogene homolog
HPI-1	Hh pathway inhibitor-1
CSC	Cancer stem cells
IL-6	Interleukin-6
NF-κB	Nuclear factor kappa-light-chain-enhancer of activated B cells
EMT	Epithelial-mesenchymal transition
Sox2	SRY-box 2
Oct4	Octamer-binding transcription factor 4
BC	Breast cancer
ER	Estrogen receptor
PR	Progesterone receptor
HER	Human epidermal growth factor receptor
DMEM	Dulbecco’s Modified Eagle Medium
FITC	Fluorescein isothiocyanate
MTT	3-(4,5-dimethylthiazol-2-yl)-2,5-diphenyltetrazolium bromide
DMSO	Dimethyl sulfoxide
PI	Propidium iodide
MMP2	Matrix Metallopeptidase 2
HCC	Hepatocellular carcinoma
FDA	Food and Drug Administration
FBS	Fetal bovine serum
NEAA	Non-essential Amino Acid
RT	Reverse transcription
PCR	Polymerase chain reaction
APC	Allophycocyanin
